# Supplementary material for: The Complete Mitochondrial Genome of Stromateus stellatus (Scombriformes: Stromateidae): Organization, Gene Arrangement, and Phylogenetic Position Within the Suborder Stromateoidei
Source: Genes (Basel). 2025 Oct 24;16(11):1256. doi: 10.3390/genes16111256 (PMC12652691; doi:10.3390/genes16111256)
Supplement: Supplementary file 1 [file genes-16-01256-s001.zip › genes-3897963-supplementary.pdf]

Table S1. Compositional organization in the mitochondrial genome of *S. stellatus*.

Region	Start position	Stop position	Length (bp)	Codon		Intergenic nucleotide	Anti-Codon	Strand
				Start	Stop			
tRNA-Phe	1	68	68	-	-	0	GAA	H
12S rRNA	69	1,018	950	-	-	0	-	H
tRNA-Val	1,019	1,090	72	-	-	27	TAC	H
16S rRNA	1,117	2,783	1,667	-	-	0	-	H
tRNA-Leu2	2,784	2,857	74	-	-	0	TAA	H
ND1	2,858	3,832	975	ATG	TAA	5	-	H
tRNA-Ile	3,837	3,907	71	-	-	-1	GAT	H
tRNA-Gln	3,907	3,977	71	-	-	-1	TTG	L
tRNA-Met	3,977	4,046	70	-	-	0	CAT	H
ND2	4,047	5,093	1,047	ATG	TAA	-1	-	H
tRNA-Trp	5,093	5,163	71	-	-	2	TCA	H
tRNA-Ala	5,165	5,233	69	-	-	2	TGC	L
tRNA-Asn	5,235	5,307	73	-	-	4	GTT	L
O_L	5,311	5,342	32	-	-	-13	-	H
O_{H_1}	5,329	5,367	39	-	-	-25	-	H
tRNA-Cys	5,342	5,408	67	-	-	0	GCA	L
tRNA-Tyr	5,409	5,475	67	-	-	1	GTA	L
COI	5,477	7,027	1,551	GTG	TAA	0	-	H
tRNA-Ser2	7,028	7,098	71	-	-	3	TGA	L
tRNA-Asp	7,102	7,171	70	-	-	8	GTC	H
COII	7,180	7,875	696	ATG	AGA		-	H
tRNA-Lys	7,871	7,944	74	-	-	4	TTT	H
ATP8	7,946	8,113	168	ATG	TAA	-10	-	H
ATP6	8,104	8,787	684	ATG	TAA	-1	-	H
COIII	8,787	9,572	786	ATG	TAA	-1	-	H
tRNA-Gly	9,572	9,643	72	-	-	0	TCC	H
ND3	9,644	9,994	351	ATG	TAA	0	-	H
tRNA-Arg	9,993	10,062	70	-	-	1	TCG	H
ND4L	10,063	10,359	297	ATG	TAA	-7	-	H
ND4	10,353	11,738	1,386	ATG	AGA	0	-	H
tRNA-His	11,734	11,802	69	-	-	4	GTG	H
tRNA-Ser1	11,803	11,870	68	-	-	4	GCT	H
tRNA-Leu1	11,875	11,947	73	-	-	0	TAG	H
ND5	11,948	13,786	1,839	ATG	TAA	-4	-	H
ND6	13,783	14,304	522	ATG	TAG	0	-	L
tRNA-Glu	14,305	14,373	69	-	-	0	TTC	L

Cyt b	14,379	15,524	1,146	ATG	TAG	5	-	H
tRNA-Thr	15,520	15,591	72	-	-	-5	TGT	H
tRNA-Pro	15,591	15,660	70	-	-	205	TGG	L
Control Region	15,866	16,454	589	-	-	55	-	H

Table S2. Length, base composition percent (account in parenthesis), GC percent, and AT and GC skew in different regions of the mitochondrial genome of *S. stellatus*.

Region	Length (bp)	A%	C%	G%	T%	GC%	AT-skew	GC-skew
ND1	975	25.87 (252)	28.44 (277)	15.09 (147)	30.60 (298)	43.53	-0.08	-0.31
ND2	1,047	27.79 (291)	33.43 (350)	12.61 (132)	26.17 (274)	46.04	0.03	-0.45
COI	1,551	25.08 (389)	25.34 (393)	18.38 (285)	31.21 (484)	43.71	-0.11	-0.16
COII	696	29.23 (204)	25.33 (177)	15.20 (106)	30.25 (209)	40.52	-0.02	-0.25
ATP8	168	30.95 (52)	34.52 (58)	11.9 (20)	22.62 (38)	46.43	0.16	-0.49
ATP6	684	28.22 (193)	31.73 (217)	12.43 (85)	27.63 (189)	44.15	0.01	-0.44
COIII	786	25.45 (200)	29.39 (231)	15.9 (125)	29.26 (230)	45.29	-0.07	-0.30
ND3	351	23.78 (83)	28.94 (103)	15.19 (53)	32.09 (112)	44.13	-0.15	-0.31
ND4L	297	24.92 (74)	32.66 (97)	14.48 (43)	27.95 (83)	47.14	-0.06	-0.39
ND4	1,386	27.9 (385)	29.93 (413)	14.57 (203)	27.61 (383)	44.49	0.01	-0.35
ND5	1,839	28.6 (526)	29.58 (544	13.54 (249)	28.28 (520)	43.12	0.01	-0.37
ND6	522	15.9 (83)	14.37 (75)	31.99 (167)	37.74 (197)	46.36	-0.41	0.38
Cyt b	1,146	25.94 (297)	28.05 (322)	15.07 (171)	30.94 (356)	43.12	-0.09	-0.30
16S RNA	950	33.83 (564	24.54 (409)	20.4 (340)	21.24 (354)	44.93	0.23	-0.09
12S RNA	1,667	31.89 (303)	25.26 (240)	21.37 (203)	21.47 (204)	46.63	0.20	-0.08
tRNAs	1,551	27.53 (427)	21.99 (341)	24.18 (375)	26.31 (408)	46.16	0.02	0.05
13PCGs	11,431	26.47 (3,026)	28.45 (3,252)	15.61 (1,784)	29.47 (3,369)	44.06	-0.05	-0.29

Control region	589	28.69 (169)	24.62 (145)	16.98 (100)	29.71 (175)	41.60	-0.02	-0.18
Whole Genome	15,509	28.79 (4,753)	27.84 (4,596)	16.32 (2,694)	27.05 (4,466)	44.16	0.03	-0.26

Table S3. Relative synonymous codon usage (RSCU) in the mitochondrial genome of *S. stellatus*. The amino acids are given in parentheses following the count and the codon frequency. Average# codons=3810. Asterisks indicate translation stop codons.

Codon	Count	RSCU	Codon	Count	RSCU	Codon	Count	RSCU	Codon	Count	RSCU
UUU(Phe)	99	0.92	UCU(Ser2)	72	1.43	UAU(Tyr)	76	0.99	UGU(Cys)	22	0.85
UUC(Phe)	117	1.08	UCC(Ser2)	70	1.39	UAC(Tyr)	77	1.01	UGC(Cys)	30	1.15
UUA(Leu2)	110	1.24	UCA(Ser2)	74	1.47	UAA(*)	32	1.23	UGA(Trp)	78	1.54
UUG(Leu2)	27	0.3	UCG(Ser2)	16	0.32	UAG(*)	31	1.19	UGG(Trp)	23	0.46
CUU(Leu1)	144	1.62	CCU(Pro)	108	1.45	CAU(His)	47	0.71	CGU(Arg)	18	0.77
CUC(Leu1)	81	0.91	CCC(Pro)	97	1.31	CAC(His)	86	1.29	CGC(Arg)	19	0.82
CUA(Leu1)	139	1.56	CCA(Pro)	73	0.98	CAA(Gln)	71	1.46	CGA(Arg)	42	1.81
CUG(Leu1)	32	0.36	CCG(Pro)	19	0.26	CAG(Gln)	26	0.54	CGG(Arg)	14	0.6
AUU(Ile)	137	1.22	ACU(Thr)	72	1.04	AAU(Asn)	73	0.89	AGU(Ser1)	15	0.3
AUC(Ile)	88	0.78	ACC(Thr)	107	1.54	AAC(Asn)	91	1.11	AGC(Ser1)	55	1.09
AUA(Met)	82	1.13	ACA(Thr)	80	1.15	AAA(Lys)	76	1.71	AGA(*)	19	0.73
AUG(Met)	63	0.87	ACG(Thr)	19	0.27	AAG(Lys)	13	0.29	AGG(*)	22	0.85
GUU(Val)	64	1.35	GCU(Ala)	73	1.03	GAU(Asp)	37	1	GGU(Gly)	48	1.02
GUC(Val)	40	0.84	GCC(Ala)	107	1.51	GAC(Asp)	37	1	GGC(Gly)	44	0.94
GUA(Val)	68	1.43	GCA(Ala)	82	1.16	GAA(Glu)	64	1.38	GGA(Gly)	58	1.23
GUG(Val)	18	0.38	GCG(Ala)	21	0.3	GAG(Glu)	29	0.62	GGG(Gly)	38	0.81

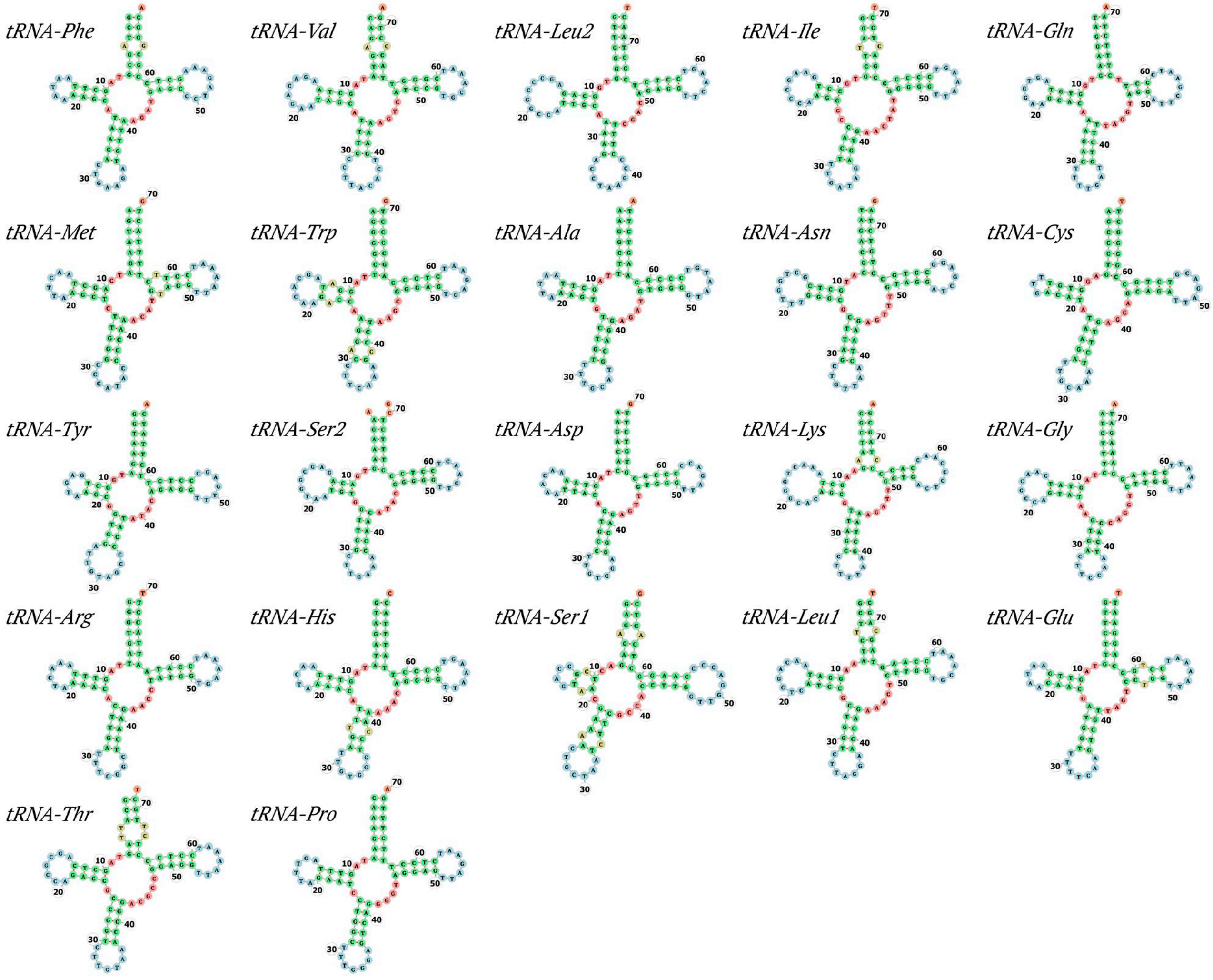

Figure S1. Secondary structure of tRNAs in the mitogenome of *S. stellatus*. Stems (canonical helices) are shown in green, multiloops (junctions) are shown in red, interior loops are shown in yellow, hairpin loops are shown in blue, and 5' and 3' unpaired regions are shown in orange.

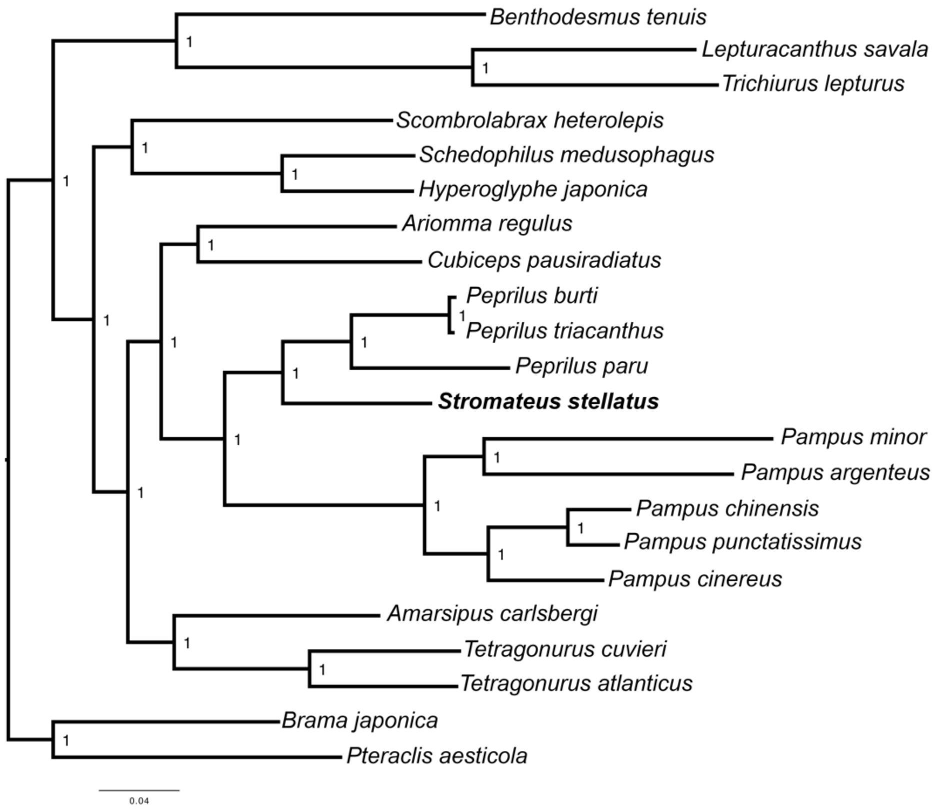

Figure S2. Bayesian inference tree of *S. stellatus* and 21 species of Scombriformes. Phylogenetic reconstruction was done from a concatenated and partitioned matrix of 13 protein-coding mitochondrial genes and 11,547 aligned sites. The numbers at the nodes represent the Bayesian posterior probabilities.
